# Novel Transabdominal Motor Action Potential (TaMAP) Neuromonitoring System for Spinal Surgery

**DOI:** 10.7759/cureus.655

**Published:** 2016-06-27

**Authors:** Reid Hoshide, Erica Feldman, Brandon C Gabel, Natalie Taylor, James Gharib, Yu-Po Lee, William Taylor

**Affiliations:** 1 Department of Neurosurgery, University of California, San Diego; 2 NuVasive; 3 Department of Orthopedic Surgery, University of California, San Diego

**Keywords:** neuromonitoring, neurophysiology, neurosurgery, spine surgery, minimally invasive, lateral access

## Abstract

**Introduction:**

Minimally invasive lateral lumbar interbody fusion (LLIF) approaches to the lumbar spine reduce patient morbidity compared to anterior or posterior alternatives. This approach, however, decreases direct anatomical visualization, creating the need for highly sensitive and specific neurophysiological monitoring. We seek to determine feasibility in 'transabdominal motor action potential (TaMAP)' monitoring as an assessment for the integrity of the neural elements during lateral-approach surgeries to the lumbar spine.

**Methods:**

Cathode and anode leads were placed on the posterior and anterior surfaces of two porcine subjects. Currents of varying degrees were transmitted across, from front to back. Motor responses were monitored and recorded by needle electrodes in specific distal muscle groups of the lower extremity. Lastly, the cathode and anode were placed anterior and posterior to the chest wall and stimulated to the maximum of 1500 mA to determine any effect on cardiac rhythm.

**Results:**

Responses were seen by measuring vertical height differences between peaks of corresponding evoked potentials. Recruitment began at 200 mA in the lower extremities. Stimulation at 450 mA recruited a reliable and distinguishable electrographic response from most muscle groups. Responses were recorded and reliably measured and increased in proportion to the graduation of transabdominal stimulation current; no responses were seen in the arms or face. 1500 mA across the chest wall failed to stimulate or induce cardiac arrhythmia on repeated stimulation, indicating safety of stimulation.

**Conclusion:**

TaMAPs seen in the animal model provide a potential alternative to standard transcranial motor evoked potentials done in the lateral approach of LLIFs. TaMAP recordings in most muscle groups were recordable and reliable, though some muscle groups failed to stimulate. Safety of transabdominal motor evoked potentials was confirmed in this porcine study. Future studies should examine TaMAPs reliability in detecting compressive lesions of nerve roots and peripheral nerves.

## Introduction

Minimally invasive lateral approaches to the spine have been shown to reduce patient morbidity compared to open anterior or posterior alternatives [1-5]. In a series reported by Rodgers et al. [6], 600 patients undergoing minimally invasive lateral lumbar interbody fusions (LLIF) had a lower incidence of infection, visceral and neurological injury, transfusion, and reduced hospital length of stays, compared to traditional open approaches. Despite its benefits, this approach had been fraught with resultant nerve injuries which were only appreciated postoperatively. The largest risk with the LLIF is iatrogenic injury to the branches of the lumbar plexus, most notably the genitofemoral nerve, which courses over the surface of the psoas muscle. The course of these neural structures can encumber the surgical corridor to the intervertebral disc space. The incidence of intraoperative nerve injury during lateral approaches to lumbar spine surgery has been shown to be as high as 30% without neuromonitoring, making this approach unappealing compared to alternative approaches to the lumbar spine [7]. The addition and advances of intraoperative neuromonitoring (IONM) have made this technique safer with lower rates of postsurgical paresis [6, 8-9].

The lateral approach to the lumbar spine has less direct anatomical visualization compared to alternative approaches. This creates the need for highly sensitive and specific neurophysiological monitoring to ensure the surgeon is not inadvertently injuring nerves that may not be readily identifiable intraoperatively. The different modalities of IONM work in concert with each other to increase the safety of surgery. One specific type of IONM is the transcranial motor evoked potentials (TcMEP) [10]. TcMEPs are carried out by electrodes placed in the scalp above the motor cortex of the brain. Stimulation of these electrodes triggers a response from the associated motor group within the homunculus. A response of the associated muscle group is recorded distally and compared to a reliable pre-incision baseline. With TcMEPs, motor stimulation is performed at every level, even for procedures involving only the lumbosacral spine, including the cranial nerves. This can cause tongue and oral mucosa biting, loss of peripheral intravenous or arterial lines with violent stimulation, and theoretical potential for cardiac arrhythmias and seizure precipitation. We seek to determine the feasibility of 'transabdominal motor action potential (TaMAP)' monitoring. Rather than transcortical stimulation, this method will employ the same mechanism of stimulation, though through selective stimulation of the lumbar plexus via a cathode placed at the anterior abdominal wall, and an anode at the posterior abdominal wall. 

In this study, we describe an animal model that analyzes the feasibility and safety of TaMAP.

## Materials and methods

This porcine study was conducted in accordance with the Biotox Science Animal Care and Use Protocol, and approved by our Institutional Animal Use Care and Use Committee (IACUC). Two porcine subjects were experimented at two different intervals. Each was anesthetized per standard protocol. The anesthetic choice was ketamine for induction, followed by isoflurane for maintenance. The anesthetic choice lacked any paralytics or muscle relaxants that would prevent accurate neuromonitoring recordings. Three posterior stimulating surface electrodes were placed over the spine from the lower thoracic to upper lumbar locations, and one anterior receiving surface electrode was placed below the umbilicus. Recording needle electrodes were placed and taped in the following positions: two pairs bilaterally to the vastus lateralis, one pair to the biceps femoris, one pair to the tibialis anterior, and one pair to the gastrocnemius medial. Stimulation parameters were set to 4 pulses, 1 msec ISI, a polarity with a 20 µVpp (microvolts peak-to-peak) response sensitivity. A TaMAP baseline was established using a threshold method for each of the three posterior electrodes, and the electrode with the lowest overall threshold was used for the balance of the procedure. TaMAP stimulation was conducted to confirm response. The current of stimulation was started at 300 mA, and then we graduated up each time to 400 mA, 500 mA, 1000 mA, and finally 1500 mA. The responses in each muscle group were recorded.

Finally, the electrodes were moved to the chest wall, with the cathode at the anterior chest wall above the heart, and the anode on the posterior chest wall between the scapulae muscles. A baseline cardiac rhythm was established and ensured that the subject was not on any vasoactive agents. A stimulus was then applied at the maximum of 1500 mA to determine cardiac safety.

## Results

### First porcine model

Our first porcine subject underwent successful anesthesia, positioning, and needle-electrode placement. We started first by obtaining TaMAP recordings in a graduated fashion as a function of stimulus current. Efferent responses were appreciable at the 200 mA level, though not reliable with the gastrocnemius and biceps femoris muscles. Multiple repositionings of the recording lead failed to elicit an accurate, reliable response. Transabdominal motor action stimulation at 450 mA recruited an electrographic response from all reliably measured muscle groups and was found to be the best current for stimulation that would cause the least muscle artifact from excessive stimulation. Muscle group responses to transabdominal stimulation proportionally corresponded to a graduated increase of the transabdominal current (Table 1). 

**Table 1:** Muscle responses in selected muscle groups with increasing stimulation currents of our first porcine subject Average muscle responses (µVpp)

Stimulation (mA)	Left Vastus (µVpp)	Right Vastus (µVpp)	Left Tibialis (µVpp)	Right Tibialis (µVpp)	Left Biceps Femoris (µVpp)	Right Biceps Femoris (µVpp)	Left Gasctrocnemius (µVpp)	Right Gastrocnemius (µVpp)
300	108	100	20	12	NR	NR	NR	NR
400	122	119	24	18	NR	NR	NR	NR
500	107	124	27	22	NR	NR	NR	NR
1000	228	771	115	28	NR	NR	NR	NR
1500	260	907	685	43	51	27	NR	NR

The left and right vastus medialis and the left and right tibialis anterior were shown to increase in proportional fashion to increased stimulation current. Responses were only seen at the level of the lumbar plexus; no responses were appreciated in the arms or face. 

Finally, we moved the anode and electrode to the chest wall to test the effects of stimulation on cardiac rhythm. A stimulus of 1500 mA across the chest wall failed to stimulate or induce cardiac arrhythmia, indicating safety of the stimulation.

### Second porcine model

The second animal underwent successful anesthesia, and TaMAPs were recorded in a similar fashion to the first animal (Table 2). Similar to the first experiment, data recording of the biceps femoris and gastrocnemius was minimally successful despite multiple attempts.

**Table 2:** Muscle responses in selected muscle groups with increasing stimulation currents of our second porcine model Average muscle responses (µVpp)

Stimulation (mA)	Left Vastus (µVpp)	Right Vastus (µVpp)	Left Tibialis (µVpp)	Right Tibialis (µVpp)	Left Biceps Femoris (µVpp)	Right Biceps Femoris (µVpp)	Left Gasctrocnemius (µVpp)	Right Gastrocnemius (µVpp)
300	123	141	35	NR	NR	NR	NR	NR
400	120	73	42	48	NR	NR	NR	33
500	134	244	84	29	84	613	NR	54
1000	193	771	188	28	791	NR	NR	NR
1500	266	869	579	43	6561	5416	338	134

**Figure 1:**
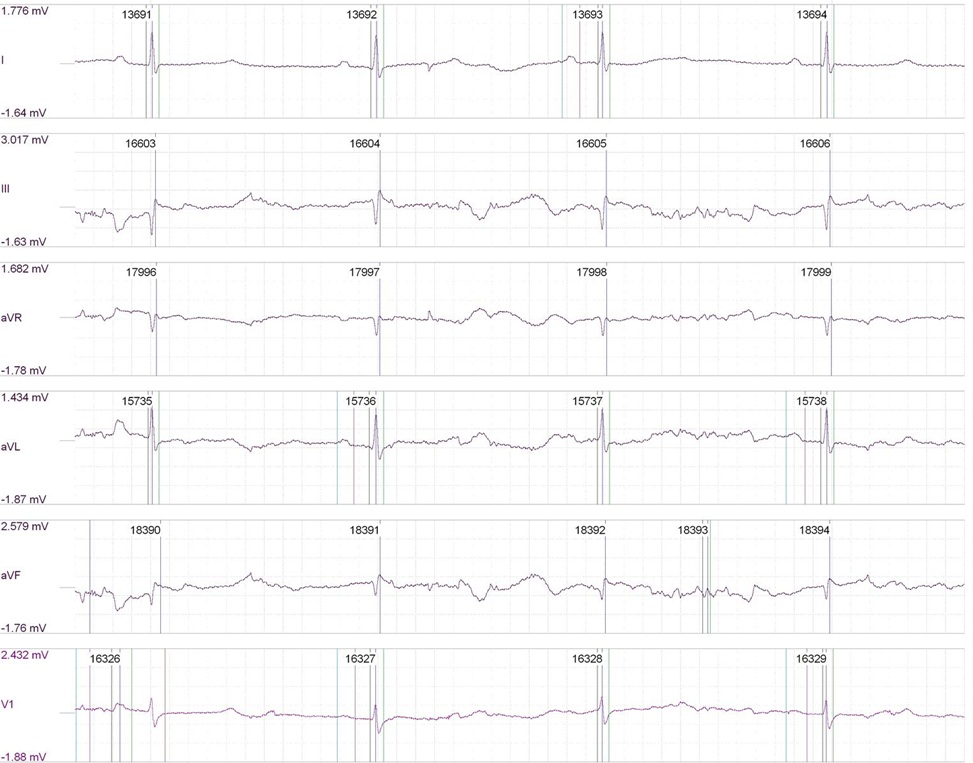
Pre-stimulation EKG of our second porcine subject

**Figure 2:**
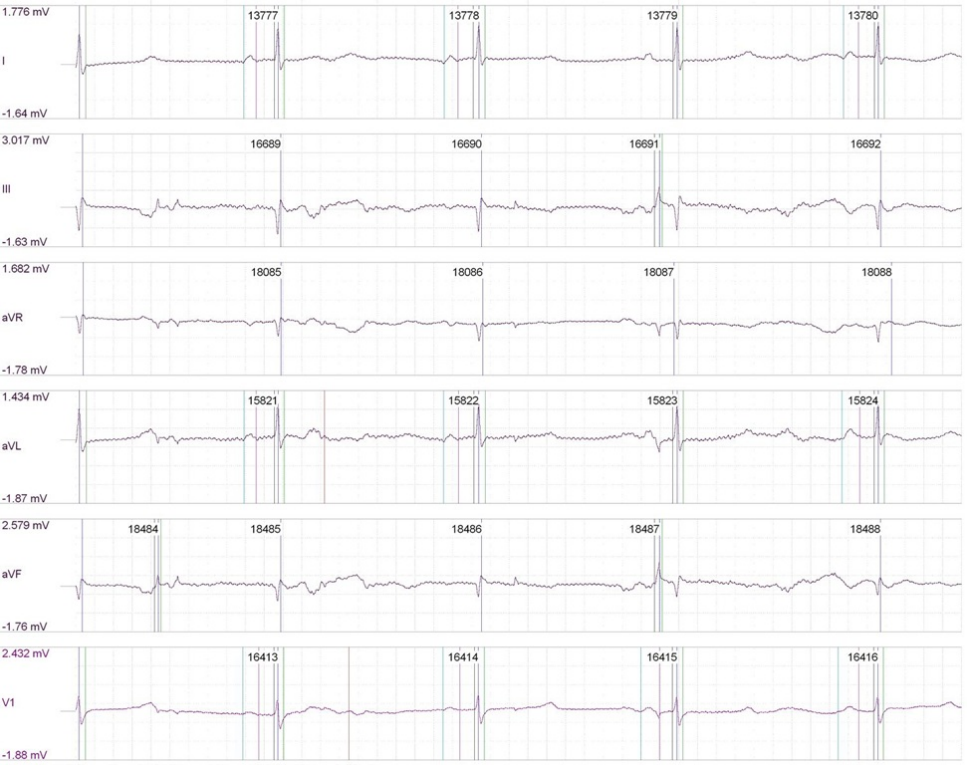
Post-stimulation EKG of our second porcine subject, showing no change in cardiac rhythm

In determining the effects of stimulation on cardiac rhythm, a stimulus of 1500 mA across the chest wall failed to stimulate or induce cardiac arrhythmia, indicating safety of the stimulation (Figures 1-2).

## Discussion

Despite the benefits of LLIF, injuries to the psoas muscle, nerve roots, or the lumbar plexus are still potential risks of the trans-psoas approach.To minimize these risks, the use of an electromyographic monitoring system during surgery has become essential in LLIF [6, 8-9].

Transcranial-evoked potentials provide important information in ensuring the integrity of the nerves at risk. However, this monitoring modality carries risks. In lumbar surgery, neuromonitoring is primarily needed at the lumbosacral levels. Limiting the stimulus to an area just proximal to the nerves of interest can reduce the unnecessary effects that may occur with concomitant upper extremity and cranial nerve stimulation [11-12]. Here, we presented TaMAPs in two porcine models. This novel method of neuromonitoring provides an intriguing potential alternative to standard transcranial motor evoked potentials for patients undergoing lumbosacral spinal surgery. 

Responses were obtained, but sometimes were hard to distinguish from large motion artifact due to very short latencies and compact, stubby morphology of the animals' extremities. The data output from the biceps femoris and the gastrocnemius muscles were plagued with motion artifact from the other tested muscle groups. Moreover, its low amplitude and unreliable recordings made it difficult to assess any true changes in the responses. It was also not feasible to fully ascertain the anatomic organization and innervation of the subject’s stubby extremities. Nonetheless, responses in selected muscle groups from transabdominal stimulation were identifiable and ascribable.

## Conclusions

TaMAP monitoring has been found to be safe in our study and avoids complications and functional limitations of TcMEPs. The current conclusions have not been proposed or tested prior to the animal model in this current study. This model confirms our proposed mechanism of function and validates the safety of the approach. Future studies should examine the reliability of TaMAP monitoring in the setting of nerve compression or injury. This novel method of neuromonitoring paves the way for future animal and human trials to improve the safety of neuromonitoring techniques.
